# Historical ecology reveals the “surprising” direction and extent of shifting baselines for the Florida manatee (*Trichechus manatus latirostis)*

**DOI:** 10.1371/journal.pone.0313070

**Published:** 2024-11-20

**Authors:** Thomas J. Pluckhahn, David K. Thulman

**Affiliations:** 1 Department of Anthropology, University of South Florida, Tampa, FL, United States of America; 2 Department of Anthropology, George Washington University, Washington, D.C., United States of America; Wrocław University of Environmental and Life Sciences: Uniwersytet Przyrodniczy we Wroclawiu, POLAND

## Abstract

Historical data are often overlooked in risk assessments and recovery plans for marine animals, resulting in the “shifting baselines.” Historical ecological studies demonstrate the “surprising” extent to which contemporary assumptions misrepresent premodern baselines. The Florida manatee, a subspecies of the West Indian manatee found primarily in Florida, USA, faces several existential threats, but risk assessments and recovery targets for the species are hindered by poor understanding of historical baselines. We conducted systematic and opportunistic reviews of archaeological and historical records of manatee occurrence in Florida, USA. Our data reveal that manatee populations in Florida were very small in the Precolonial and Colonial Periods, possibly representing infrequent in-migration from the Caribbean during favorable climate conditions. Manatees expanded in number and range across the Florida peninsula during the Territorial/Early Statehood and Early Modern Periods, first northward on the Atlantic Coast and later along the coast of the Gulf of Mexico. These expansions track increasing human populations, associated anthropogenic landscape alterations, and social and policy changes. Historical ecology is critical for “shaping a better Anthropocene” for humans and manatees in Florida.

## Introduction

The past few years have been challenging for the Florida manatee (*Trichechus manatus latirostis*), one of two subspecies of the West Indian manatee. Almost fifty years after listing as “endangered” under the Endangered Species Preservation Act in 1973, the manatee was downlisted to “threatened” by the U.S. Fish and Wildlife Service (FWS) [1]. However, recent upticks in manatee deaths on the Atlantic Coast led the FWS and the Florida Fish and Wildlife Conservation Commission (FWC) to declare an “unusual mortality event” for that region, which remains ongoing [2]. Beyond longstanding threats from collisions with watercraft and cold stress, the Florida manatee faces several newer existential challenges: loss of warm-water refuges with the closure of older fossil-fuel power plants (as well as new legal limits on effluents) [3–5], loss of forage with the proliferation of algal blooms [6], and loss of habitat owing to climate change [7].

Historical data are often overlooked in risk assessments and recovery plans for marine animals [8], resulting in the “shifting baselines” phenomenon (Pauly 1995:430). A number of historical ecological studies have demonstrated the problems with perceptions of historical baselines, sometimes revealing the “surprising” extent [9] to which they underestimate the loss of marine taxa in the modern era [10–14]. Baselines are also known to shift in the opposite direction; for example, Baisre [15] demonstrated that the Caribbean monk seal (*Monachus tropicalis*) exhibited a small and fragmented population in the 1500s, contrary to assumptions the species was formerly abundant and widespread [16].

The extent of human-induced changes to species assemblages and ecosystem functioning in the Anthropocene has prompted some to question the relevance of historical baselines for conservation and management decisions [17–20]. Kopf and colleagues [21] propose the concept of “Anthropocene baselines” to recognize that the rehabilitation capacities of some “novel ecosystems” [22] are so compromised that new points of reference are needed to manage them. While the concept of ABs has been framed as a “shift away from the constraints of historical references” [21], the management of novel ecosystems still requires data-supported estimates of historical ranges of variation in species abundance and distribution.

The range and population of the Florida manatee were poorly understood before the 1950s, when Moore [23–25] began pioneering surveys based on his observations and those of informants. Formal ground and aerial surveys began in the late 1960s [26–30]. Only a few studies have considered evidence for pre-modern manatee populations. Cumbaa [31], based on a review of archaeological evidence, concluded that Precolonial sea cow populations were too low “to constitute a harvestable, renewable resource” and that Indigenous exploitation of manatee was “negligible.” O’Shea [32], drawing from Cumbaa and a review of historical sources, suggested that a “long history of hunting pressure…probably always suppressed manatee populations” preventing them from becoming “extremely abundant” but never leading to their near extinction. These widely cited but relatively brief and unsystematic reviews, coupled with an absence of follow-up studies, underlie the assumption that manatees were as (or more) numerous and widespread as they are today before human predation [3, 5, 30, 33–37].

**Fig 1:**
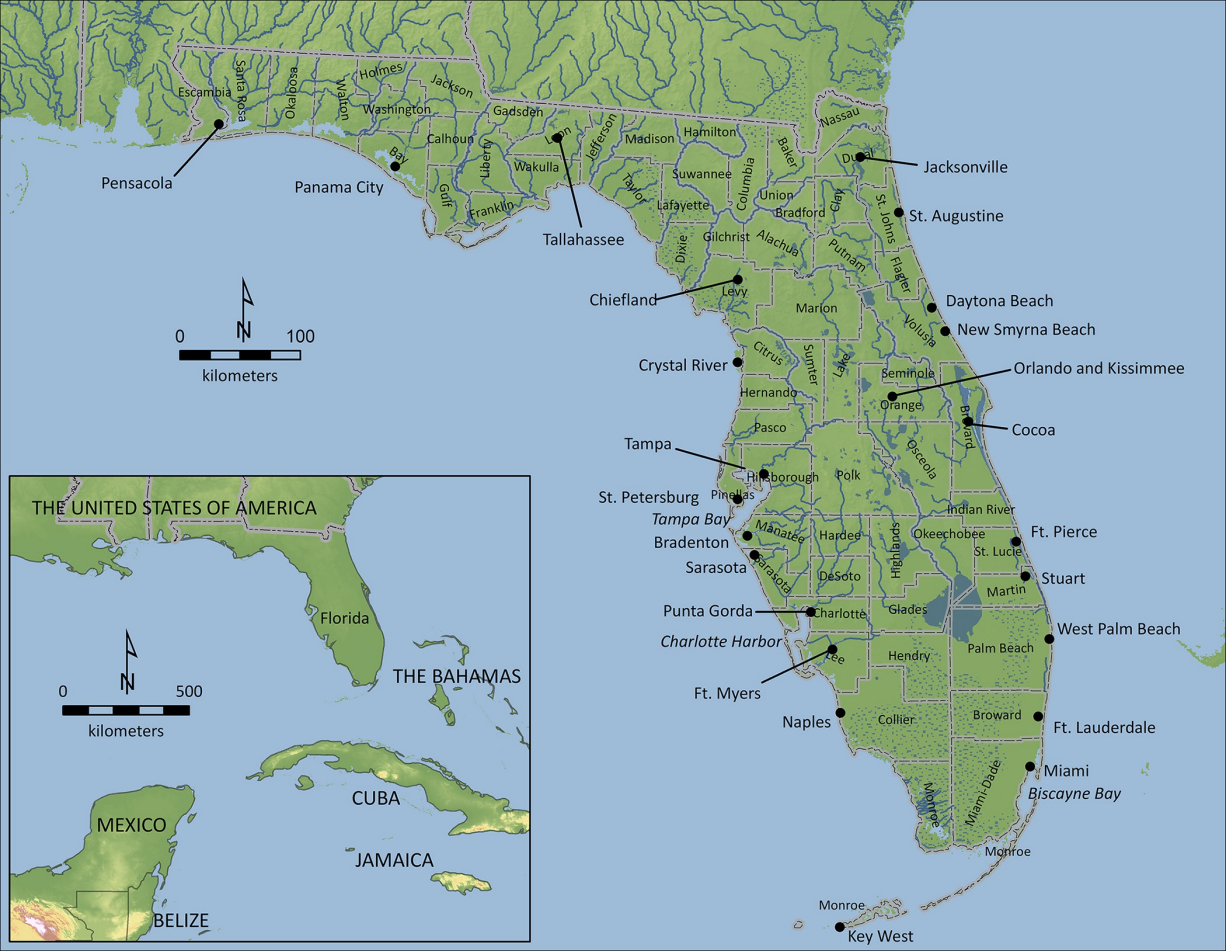


We conduct a more thorough and systematic evaluation of archaeological and archival evidence for the variation in the abundance and range of manatees in Florida (Fig 1), from the time of initial human settlement 14,000 years ago to the late 1950s. Our review confirms that manatees are sparsely represented in archaeological and historical records of the Precolonial (12,000 BCE to 1513 CE) and Colonial (1513–1822) periods, became more numerous in southern Florida during the Territorial/Early Statehood Period (1822–1860), and expanded in number and range in the Early Modern Period (1860–1960). We identify several factors to explain these patterns: natural and anthropogenic climate change (revealed by a review of previous paleoecological studies and a compilation of modern climate records); increasing human populations (indicated by census data); and associated anthropogenic alterations of the landscape as well as social and policy changes (revealed through historical sources).

## Materials and methods

Our analysis of manatee population and range focused on two data sources: reports of the occurrence of manatee remains on archaeological sites from the precolonial and historic eras and archival reports of manatee sightings. For each, we include systematic and opportunistic samples.

Our systematic sample of archaeological reports was structured in two ways. First, we searched the online database of the Florida Master Site File for reports of archaeological testing and excavation, which are more likely than survey reports to produce vertebrate faunal assemblages. Next, we limited the sample to testing and excavation reports that included systematic analysis and reporting of vertebrate faunal remains. When we identified a report that met these parameters, we recorded the total number of individual specimens (NISP) (i.e., fragments of bone) identified for the total assemblage and for manatees. We also recorded the minimum number of individuals (MNI) (i.e., the least number of individuals of each taxon, based on counts of repeating elements), in total and for manatees. This sample included 67 reports, which record a total vertebrate faunal NISP of 1,988,797 and a total MNI of 29,735 (see S1 Dataset, Systematic Archaeology).

Our opportunistic archaeological sample included other reports of archaeological investigations—identified through a general literature review and through the systematic search described above—where the occurrence of manatee bones was described separately from systematically analyzed faunal assemblages. This sample consisted mainly of reports of manatee bones modified into tools or ornaments (generally reported separately from unmodified faunal remains) as well as unmodified bones tentatively identified as manatee or possibly manatee without a formal zooarchaeological analysis. Our opportunistic sample included 12 reports of manatee or possible manatee remains from archaeological sites (see S1 Dataset, Opportunistic Archaeology).

For archival sources, our opportunistic sample included published accounts of Florida manatees from the Colonial through Early Modern Periods, excluding newspapers (for reasons described below) but including the accounts of early travelers and naturalists, as well as other printed sources such as magazines, postcards, photographs, and children’s books. Our opportunistic sample included 57 descriptions of Florida manatees published between the 1500s and 1959 (see S1 Dataset, Opportunistic Archival).

Our systematic archival sample focused on newspaper accounts identified by searching newspapers.com^TM^ for Florida newspapers containing the words “manatee” or “sea cow,” resulting in 455 news reports of manatee sightings between 1867 and 1960 (see S1 Dataset, Archival Systematic). This sample was systematic in terms of our search parameters but not with regard to the reporting of manatee sightings. However, across the period of our sample, the sighting of manatees was newsworthy, often reported on the front page (e.g., [38]), and even after sightings became more common they were regularly reported by fishing columnists (e.g., [39]. We summarize the manatee sighting data as counts by county but note that boundaries and names have changed over the period of our study. Separately, we also recorded reports of applications for permits to kill or capture manatees.

Florida manatees experience cold stress syndrome (CSS) from prolonged exposure to water below 20°C [40, 41]; the FWC uses consecutive days with air temperatures ≤9.5°C as a benchmark for predicting manatee aggregation in warm-water refuges [42, 43]. To consider the frequency of manatees relative to changes in climate in the Early Modern Period, we compiled the number of daily lows and daily averages ≤9.5°C for the period from the late 1800s to 1959 from the records of the National Centers for Environmental Information (NCEI) [44]. We focused on five Florida cities, but because few stations have complete records, we sometimes combined nearby stations to provide better temporal coverage; our sample includes the following cities/stations: Ft. Myers; Ft. Pierce/Stuart; Tampa/Tampa International Airport; Orlando Executive Airport/Kissimmee City Hall; and Jacksonville/Jacksonville Naval Air Station. We present data for years for which there were at least 360 records.

We also correlate changes in manatee number and range relative to human population growth and power production. For the former, we employed U.S. census data compiled by the Florida Center for Instructional Technology, University of South Florida (FCIT) [45] and normalized these by county area. For power production, we relied on summaries of the number of plants and their production produced by the Department of Commerce and Labor, Bureau of the Census (DCL) [46–51].

## Results

### Precolonial Period (ca. 12,000 BCE-1513 CE)

More than 35 species of the Order Sirenia have existed over the last 55 million years, but only four species in two families (Trichechidae and Dugongidae) are still extant [52]. Sirenian fossils are common in Florida Oligocene (35–23 mya) and Miocene limestones (23–5 mya), when much of Florida was under shallow seas. Fossil Trichechids of Pleistocene (2.5 mya-12,000 ka) age are known from several Florida localities [53–56]. However, it seems doubtful that manatees could have survived the periodic episodes of cold air and meltwater that persisted along the Florida peninsula as late as 14,000 ka [57]. More likely, the Florida manatee developed from Caribbean stock that migrated north after the last glacial episode [3, 52, 58], consistent with limited genetic diversity suggestive of a relatively recent founder effect [52, 59–61].

Native Americans began living in Florida near the end of the Pleistocene [62], during the Paleoindian period (ca. 12,000 to 8,500 BCE). Several manatee fossils of presumed Late Pleistocene age have been interpreted as showing signs of human predation or butchering [3, 30, 31, 63]. However, none are from secure archaeological contexts. Manatees are not among the vertebrate taxa (including mastodon, camelids, and bison) recovered from excavations at the Page-Ladson site [62, 64] or at other Paleoindian archaeological sites with Late Pleistocene fauna [65–67].

No manatee bones were identified in 54 reports of investigations at sites associated primarily or exclusively with the Precolonial Period in our systematic archaeological sample, which yielded a total NISP of 1,843,775 and MNI of 26,397. This absence is striking considering the presence of several unusual taxa, such as Cetacean (whale or dolphin) (e.g., [68, 69]McLean 2019; Mikell 2012), Florida panther (*Puma concolor*) (e.g., [70, 71]), and Caribbean monk seal (e.g., [72, 73]). Even the Great Auk (*Pinguinus impennis*), a flightless bird restricted mainly to the Northern Atlantic before going extinct in the mid 1800s, is better represented than manatee in our systematic archaeological sample [68]; see also [74, 75]).

**Fig 2:**
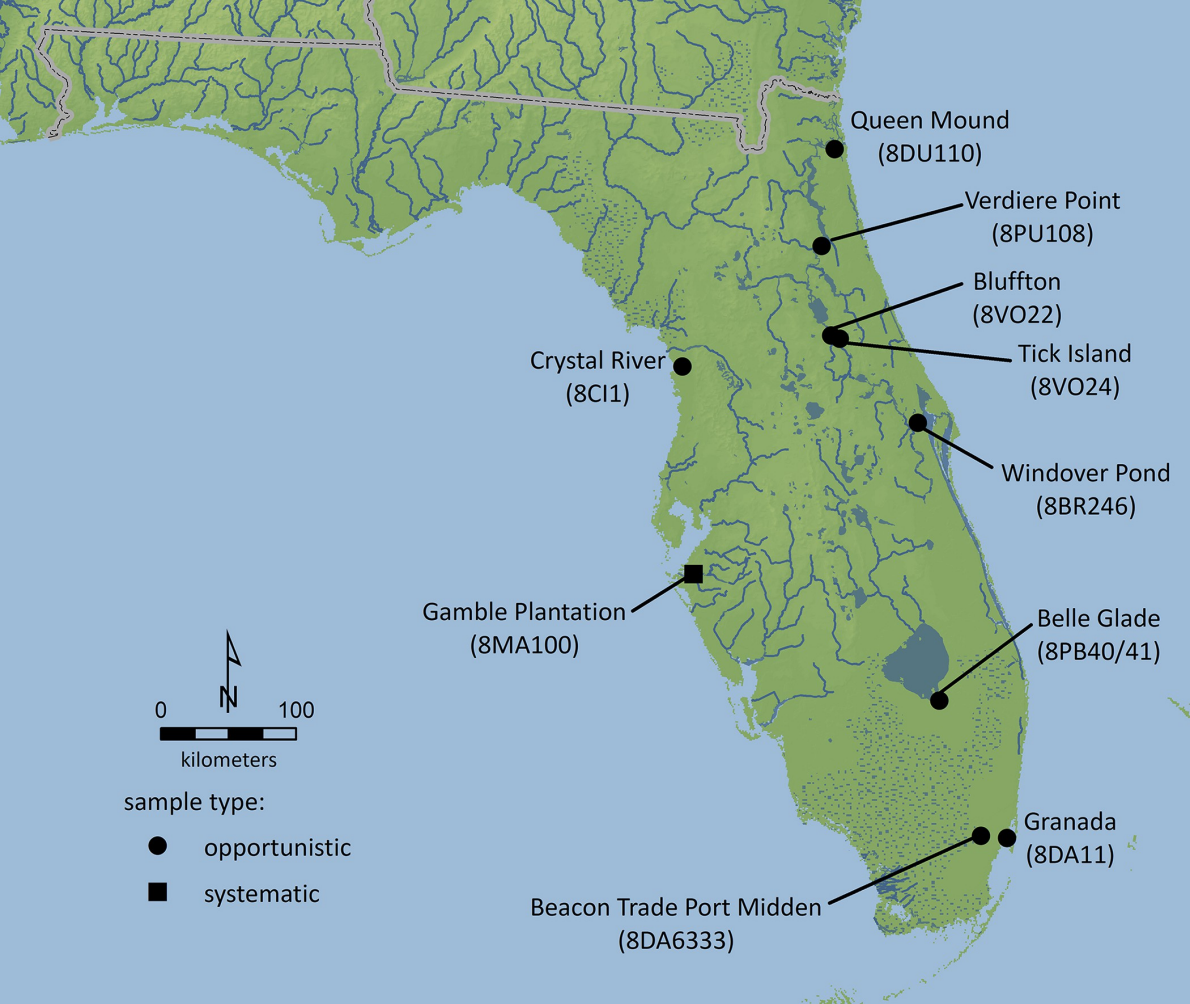


Our opportunistic archaeological sample included claims for manatee bones or fossils from nine sites dating to the Precolonial Period (Fig 2). The earliest consisted of an unfossilized rib bone modified as an atlatl (spearthrower) weight found in the comingled burial of subadults at the Windover site, an Early (8000–6000 BCE) to Middle (6000–3000 BCE) Archaic period mortuary pond [76].

Manatee remains are reported for two Late Archaic period (3000–1000 BCE) sites. Bullen [77] described rib fragments from the Bluffton site but noted these were fossilized and likely scavenged by Native peoples; Neill et al. [78] noted no manatee in their analysis of faunal remains. Cumbaa [31] described manatee bone from Tick Island, but this identification is not confirmed by Jahn’s [79] faunal analysis.

Manatee are reported at four or five sites dating to the Woodland period (1000 BCE to 1000 CE), although these are tentative identifications and some lack secure provenience (making it difficult to say precisely how many sites are represented). At the Belle Glade site, Willey [80] identified a “large paddle-shaped implement” manufactured from the scapula of a large animal, “possibly a manatee”; Willey [80] also listed manatee among the faunal remains but provided no quantities or contexts. A “possible” manatee bone was found at the Beacon Trade Port Midden [81]. Thulman [82] tentatively identified a fossil bone tool from the Queen Mound as manatee, based on a photograph [83]. Finally, there are reports of possible manatee bones and a bone tool at or near the Crystal River site [31, 84, 85].

Two manatee rib bones modified for use as plummet-pendants are reported for the later Mississippian period (1000–1500 CE) at the Granada site [86]. The report does not indicate whether the bones were fossilized.

### Colonial Period (1513–1822)

Spanish and French exploration and settlement of Florida began in the early 1500s; over the next three centuries, the colony went from Spanish (1513–1763) to British (1763–1783) rule, and back to Spain (1783 to 1822). Colonial written records are plentiful yet contain few unambiguous references to manatees.

De Herrera y Tordesillas [87], in his 1615 account of de Leon’s 1513 expedition to Florida, described sighting “lobos marinos” (“sea-wolves”) in the Tortugas. In the 1550s, Fontaneda [88] used the same term to describe animals eaten by Native peoples of southern Florida. Some translators have equated “sea-wolves” with manatees [89, 90], but others suggest the term refers to seals [25, 91]. Similar problems in translation plague Fontaneda’s description of hunting “vallena”; Swanton [92] repeats a translation of this as “sea cow,” but modern scholars prefer “whale” [25, 93–95].

Manatees are not described in accounts of the expeditions of de Narváez [96] or de Soto [97], which landed in Tampa Bay in 1528 and 1539 (respectively). Nor are manatees mentioned in the account of de San Miguel [98], who lived among the Indians in northeastern Florida for several months in 1595. Likewise, Laudonnière [99] does not mention manatees in the chronicles of his voyages to northeastern Florida in 1562, 1564, and 1565.

Colonial accounts of Florida from the 1600s also fail to mention manatees. Dickinson [100], who was shipwrecked on the southeastern coast of Florida in 1696, makes no mention of manatees despite noting much of the food provided by Indigenous peoples. Likewise, there are no references to manatees in the Spanish colonial records of St. Augustine, although there are references to the extraction of “amber” (ambergris) from “fish” (whales) [101]. Secondary summaries of Spanish records from the 1600s and 1700s also do not mention manatees [94, 95, 102].

The first reliable written accounts of manatees in Florida date to the period of British rule in the late 1700s. In 1763, Robinson [103]observed that: “of fish on their coasts, and in the bays, they have the paracod, tortoises of five several kinds, manatee, &c.” In 1774, Bartram [104] observed the skeleton of a manatee at “Manate [sic] Spring” along the Gulf Coast near modern-day Chiefland that had been killed by Indians the previous year. We are less confident in the taxonomic accuracy of a 1764 petition to the British Crown to restrict grants of land “at or near the point called the Cape of Florida…resorted to by the animals called manati or the Sea Cow” [105] (British Colonial Office 5/563, East Florida Records, 20 Jan to 24 Dec 1764, *Colonial America*); in the 1700s, British colonials sometimes referred to seals as “sea cows” (e.g., [106]) and the accompanying description of “the quantity of oil they produce” and “their Echouries” (a French-Canadian term for pinniped landings) seem more consistent with seals. Accounts of Florida in this era by Gauld in 1765 [107], Romans in 1775 [108]; de Evia in 1783; and Folch y Juan in 1793 [109], lack any mention of manatees.

Archaeological data corroborate the scarcity of manatees through the Colonial Period. Our systematic archaeological sample includes no manatee among a total NISP of 133,547 and total MNI of 3017 from more than ten sites associated with Spanish colonial settlement in St. Augustine, Pensacola, and Tallahassee, as well as British colonial settlement in New Smyrna.

### Territorial and Early Statehood Period (1822–1860)

Florida became a territory of the United States in 1822 and a state in 1845. Manatees are mentioned several times in travel accounts and scientific works from this period. Although mostly secondhand, these suggest manatees became more common in this interval, at least in southern Florida. In 1825, Harlan [110] described manatees as being “found in considerable numbers…near the capes of East Florida.” Audubon [111], on a visit to the Keys in 1832, noted that his pilot had been employed in hunting “those singular animals called Sea Cows or Marratees [sic], and he had conquered hundreds of them…because the flesh and hide bring ‘a fair price,’ at Havannah [sic].”

The few historical accounts of manatees for the Gulf Coast for this interval indicate they were present but not numerous. In an 1822 survey of Tampa Bay, Seymour [112] wrote: “There is also a fish (that I did not see) called the Manattee [sic], which is of great value…its fat always commands in Havana, the same price as our purest lard; its body is highly esteemed when salted." His claim is substantiated by the appearance of the toponym “Manatte” [113] or “Manaties” [114] in 1776 and 1809 (respectively) in reference to a river in Tampa Bay, although later accounts suggest the name derived from the “bleaching [possibly fossilized] bones” (e.g., [115, 116]). However, McCall [117], who hunted and fished extensively during his military service in Tampa Bay from 1824 to 1846, never mentioned seeing a manatee.

We identified only two archaeological sites from this period with systematic vertebrate samples, with a total NISP of 7800 and MNI of 249. No manatees are represented. However, there is one report of manatee in our opportunistic sample; Wayne and Dickinson [118] describe the recovery of fragments of manatee bone from the remains of a structure associated with enslaved Africans at the Verdiere Point site. The report does not indicate if these were fossilized, but a mastodon tooth was also recovered.

### Early Modern Period (1860 to 1960)

Our systematic archaeological sample includes the identification of manatees from one context dating to the Early Modern Period, from late nineteenth-century contexts at Gamble Plantation on the southern shore of Tampa Bay [119]. However, the bones were fossilized and thus likely not representative of the contemporaneous occurrence of manatee.

Although variable, historical accounts in our opportunistic archival sample generally agree that before the middle twentieth century, manatees were most common along the Atlantic Coast of the southern and central Florida peninsula, especially the St. Lucie, St. Sebastian, and Indian Rivers (e.g., [120–123]. Several in this region were reported killed by a hard freeze in the winter of 1894–95 [124].

In his 1896 memoir, Cory [125] recalled having “often accompanied Osceola^1^ and other Indians on a manatee hunt” and suggested that the Indians killed “many of these animals…every year.” A recent review of historical accounts of Seminole foodways by Gonzalez [126] did not corroborate Cory’s account, but photographs from the early 1900s document manatee hunting by Seminoles. In addition, tribal members recall times when manatees were hunted out of necessity (e.g., [127, 128].

Archival accounts from the late 1800s and early 1900s describe manatees as found only “sparingly” [129] or “sporadically” [130] along the Gulf Coast, mainly south of Tampa Bay [120] in Charlotte Harbor [125] and rarely to the north in the panhandle [122]. This pattern is consistent with Moore’s [23, 24] later observation that manatees were particularly abundant in two locations in southern Florida: Biscayne Bay (specifically urban and suburban Miami) and the western portion of Everglades National Park (ENP). Moore [23] cited informant reports suggesting manatees were relatively common on the Atlantic Coast north to St. Augustine but scarce on the Gulf Coast north of Charlotte Harbor.

**Fig 3:**
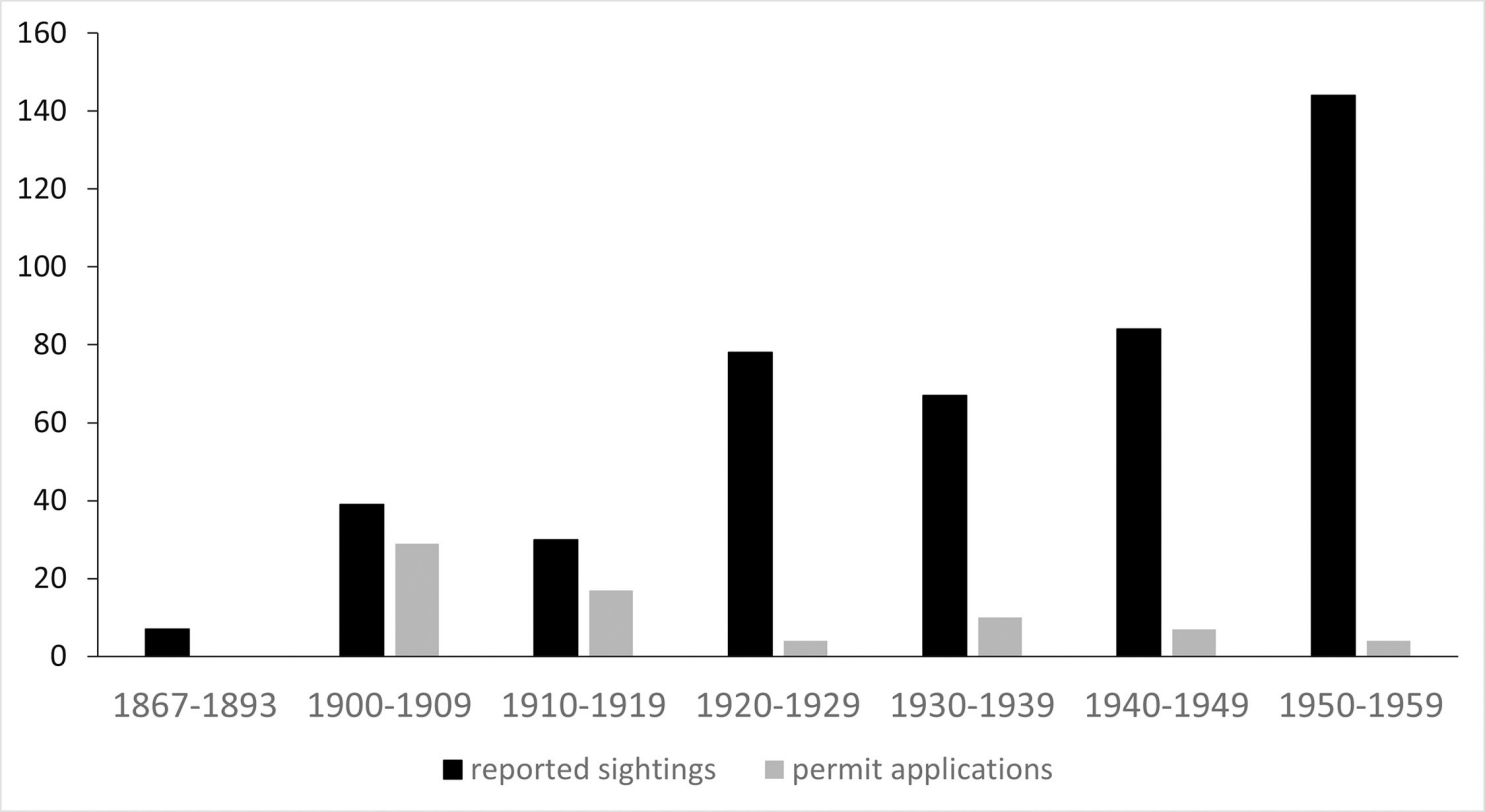


**Fig 4:**
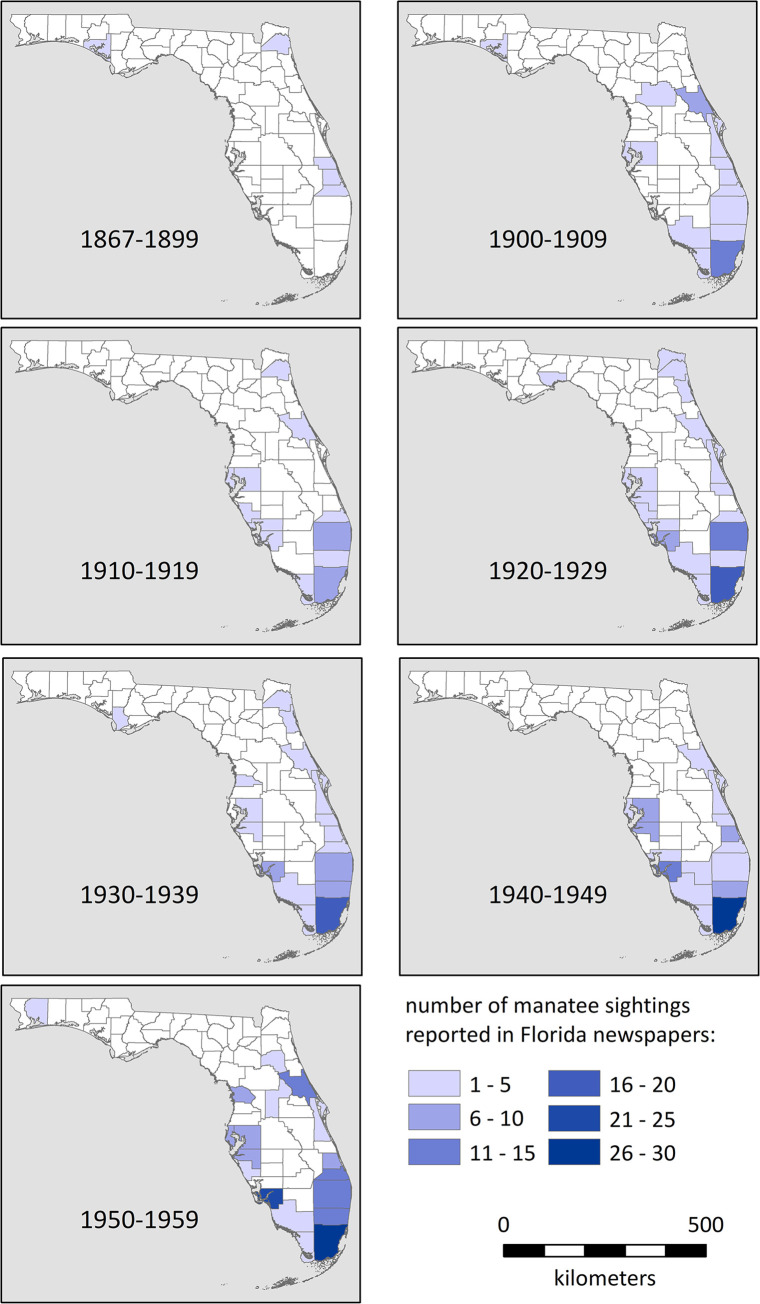


Our systematic archival sample confirms and refines these anecdotal reports. Through the late 1800s, reports of manatee sightings were uncommon in Florida newspapers (Figs 3 and 4). This reflects a paucity of news coverage relative to later periods, but the fact that manatees were rare is evident by the public spectacle that resulted when one was spotted; for example, when a dead manatee washed ashore in the western panhandle, *The Daily News* [131] of Pensacola reported that it “will no doubt attract thousands of spectators as soon as its presence becomes generally known.” The scarcity of manatees fueled speculation that they must have been more plentiful before overhunting (e.g., [132, 133]).

The southeastern Atlantic Coast may have been an exception. In 1891, writer Kirk Monroe asserted that the manatee “abounds” in Biscayne Bay and related an incident in which his Miami neighbors killed one of a “herd” of five, supplying the “settlement of 100 souls with meat for three days” [134]. Manatees were captured in the Indian River in 1888 [135] and the St. Lucie River in 1890 [136]. Manatees were occasionally reported to the north, but only when they washed up dead, as in Jacksonville in 1891 [137] and Mobile in 1893 [131].

In 1892, a company filed a charter to raise manatees in Biscayne Bay for the production of meat and skins [138]^4^, a proposal which may have motivated a bill to protect manatees [139]. Some articles suggested that the law, enacted the same year, had an immediate effect; in 1895, *The Ocala Evening Star* [140] reported that owing to its passage, the “manatee of south Florida is rapidly multiplying, and it seems that the fear…that this curious aquatic animal would soon become extinct, is groundless.” While the law was not the only factor, there were many more reports of manatees in the first two decades of the 1900s, with sightings especially common in Dade County and northward along the Atlantic Coast. In 1906, a Miami newspaper [141] described manatee sightings as “almost an everyday occurrence” near the city’s docks.

Manatees were rare on the Gulf Coast through the first two decades of the 1900s. In 1912, fishermen in Sarasota County caught and exhibited a manatee, attracting “over 200 people” [142]. A dead sea cow and calf in the Anclote River attracted “hundreds of curious humans and hungry buzzards” [143]. Longtime fishers and ferry boat captains often described the sighting of a manatee as their first, or the first in the area in many years (e.g., [144, 145]).

Newspaper articles from the 1920s and 1930s document routine sightings of manatees along the length of the Atlantic Coast, with aggregations of a dozen or more reported from Miami (e.g., [146]) to St. Augustine (e.g., [147]). In 1939, the fishing reporter for a Miami newspaper estimated the manatee population at around 600 [148]. Although no large aggregations were reported for the Gulf Coast, articles routinely described sightings of one or more manatees, especially in Charlotte Harbor (e.g., [149]). Reports of manatee sightings in Tampa Bay increased to a lesser extent and the sightings were often met with surprise (e.g., [150, 151]) or skepticism (e.g., [152]).

By the 1940s, there were reports of manatees across most of Florida’s peninsula. In 1941, The *Ft*. *Lauderdale Daily News* [153] claimed manatees “may be seen now in constantly increasing numbers.” In 1953, Pennekamp [154] wrote that one could observe a dozen “at any time” near the Miami Ave. bridge in Miami. By the 1950s, the arrival of manatees to Blue Springs, north of Orlando, was reported as a harbinger of cooler weather and became a tourist attraction (e.g., [155]). Manatees were also an attraction for visitors to ENP (e.g., [156]).

Still, several anecdotes indicate that the manatee’s distribution remained patchy well into the twentieth century. In 1953, when the owners of an aquarium in the Keys petitioned Monroe County to house a manatee, they noted the need to seek one in the Everglades given local scarcity [157]. Similarly, officials in Manatee County were unable to find one of their namesake animals locally for the annual De Soto celebration in 1948 [158]; the festival went on without one, but a manatee was obtained the following year from a Miami aquarium [159].^2^ By the mid-1950s, however, there were reports that manatees were “becoming more plentiful” in Tampa Bay [39] and a few were said to have become “permanent residents” of Crystal River [160].

## Discussion

For the more than 13,000 years of Indigenous Precolonial occupation of Florida, we have been able to identify claims for the identification of one or more manatee bones from nine archaeological sites, five of which are problematic owing to lack of provenience or secure taxonomic identification. Two more are more securely identified taxonomically, but the bone is fossilized so may not be contemporaneous with human interactions. We consider five possible explanations for why manatees are so poorly represented in the archaeological record of Precolonial Florida.

The first, and we think most likely explanation is that manatees were not present in anything close to their current numbers or extent. One possibility is that manatees were not present at all and these few specimens represent material acquired through trade with Indigenous peoples elsewhere in the Caribbean. The fact that the better-documented specimens from Precolonial archaeological contexts had been fashioned into tools or ornaments may support this conjecture; although Precolonial Native peoples of Florida also traded raw materials (e.g., exotic stones, metals, minerals), most of the exchange goods recovered from archaeological contexts were modified into forms such as these (e.g., [161, 162]). We also note that the form of the “paddle-shaped implement” from the Belle Glade site [80] resembles vomiting spatulas made of manatee bone from Taino sites in the Caribbean (e.g., [163, 164]). However, claims for direct connections between Florida and the Antilles based on artifact similarities have been rightly criticized for lack of rigor [165–167].

A second, related possibility is that manatees only occasionally migrated north from the Caribbean, as they are known to do today [168, 169], when climate conditions favored range extension. Although the sample is small, there is an increase in manatee representation over time from the Archaic to Woodland periods, consistent with the onset of warmer and wetter climate regimes, sea level stabilization, and the development of more productive estuaries [170–172], as well as increasing focus on marine and estuarine resources by Indigenous peoples and their settlement in communities of greater size and permanence [161, 173]. The lesser frequency of manatee on later precolonial (Mississippian) sites could reflect a reduction in northward manatee migrations owing to the Little Ice Age (LIA), a period of intermittent cooling beginning in the 1200s [174, 175]. The effects of the LIA in Florida are poorly understood, but isotope analyses of planktonic foraminifera from the northeastern Caribbean suggest sea surface temperatures (SST) were sometimes around 2°C cooler in winter [176]. Manatees today occasionally migrate south to Cuba [177] and may have done the same in the past as cooler conditions became a threat.

A third, and also related, possibility is that Precolonial manatee populations approximated those of today but were more diffusely dispersed over a broader range of habitats, rendering them so sparse that they were infrequently hunted by precolonial peoples. We find this hypothesis unlikely for several reasons. First, the Native precolonial population of Florida was also widely dispersed; with the possible exception of a few extremely xeric ecosystems, such as the Sand Pine Scrub at the center of the peninsula [178], there are few areas lacking archaeological evidence for intensive Native American settlement at some point in the Precolonial era. Further, precolonial Native American settlement generally favored areas in proximity to coastal estuaries and interior rivers and springs (e.g., [179]), the same habitats favored by manatees and locations which are well represented in our archaeological sample. We also reiterate that several taxa that were undoubtedly either rare or widely dispersed in Florida, such as the Great Auk and Florida panther (respectively), are better represented in our systematic archaeological sample than manatee.

A fourth, alternative hypothesis is that manatees were present in greater numbers and hunted more commonly than the archaeological record suggests, but recovery is biased by past Indigenous processing and disposal practices. For example, Native peoples of Nicaragua have historically observed a tradition of returning the bones of manatees to the water after butchering to ensure future hunting success (e.g., [180]). However, even under the unlikely assumption that similar disposal traditions held across the entirety of Native peoples of Florida for thousands of years, we would expect more manatee bones to have been recovered from the more than 40,000 archaeological sites recorded in the state.

A fifth and final potential explanation is that manatees were present in numbers comparable to today but were rarely hunted by Precolonial peoples, because they were too difficult to kill [78] or were considered sacred or taboo.^3^ However, there is abundant evidence of manatee exploitation by Precolonial Indigenous peoples elsewhere in the circum-Caribbean, from coastal Mesoamerica [181–183] to the Antilles [164, 184–189]. In some of these areas, manatees were hunted despite—or perhaps because—they were perceived as having special properties (e.g., [187]). If manatees held similar importance for precolonial peoples of Florida, we would expect them to be represented in symbolic artifacts, but there are no definitive representations of manatees in the extensive range of zoomorphic figures (including dolphins and whales) identified in wood, stone, bone, shell, and ceramic art [190]. ^4^

If manatee populations in the Precolonial period approximated modern numbers and range, we would expect their mention in the earliest Colonial Period records, mirroring accounts from the Caribbean and South America [191]. However, none of the earliest colonial accounts of Florida contain unambiguous references to manatees, despite colonists’ need for provisions and curiosity regarding Indigenous hunting and fishing technologies. For example: de Soto’s men spent time fishing in Tampa Bay [192], de San Miguel [98] described Native whale hunting, and Laudonnière [99] described many varieties of fish, fish weirs, and shrimp nets used by Florida’s Indigenous peoples.

Nor is there archaeological evidence for manatees in the extensive faunal assemblages from St. Augustine or other Spanish colonial sites in Florida. Based on evidence from elsewhere in the Caribbean, Spanish colonists would have killed and eaten manatees had they been present; Baughman [191] cites sixteenth-century sources indicating that the Catholic church considered manatees as fish, thus permitting their consumption on Fridays. Manatee remains have been identified from the sixteenth-century Spanish sites in the Dominican Republic [193] and Haiti [194].

The lingering effects of the LIA may explain the continued absence of manatees into the Colonial period. Accounts of the de Soto expedition suggest the climate of the Southeast U.S. was cooler in the middle 1500s [195, 196], and Spanish missionaries described freezing conditions in Florida in the later sixteenth century [196]. Isotope studies of corals and ostracods suggest SSTs in the Caribbean were 2–3 C° lower during the LIA of the 1700s and 1800s, although the precise timing of the coldest intervals is unclear [197–199].

Historical accounts and limited archaeological evidence indicate that manatees were present in Florida in the late 1700s and early 1800s. Bartram’s observation of a dead sea cow in a spring on the northern peninsular Gulf Coast is an exception, as written accounts from the late 1800s and early 1900s consistently describe manatees as more common along the southeastern Atlantic coast. Newspapers confirm this pattern and document the expansion in manatee numbers and range, up the Atlantic Coast in the early 1900s and northward along the Gulf Coast as far as Crystal River by the 1950s. We suggest six major factors contributed to these increases in numbers and range across the Modern era.

The 1893 state law protecting manatees was one important factor, although illegal manatee hunting continued, as described by Cory [125] and advertised for tourists in northern papers [200] in 1896. Legal hunting of manatees also continued owing to an exemption allowing capture or killing for scientific purposes under permit from county commissions, a provision widely exercised in the early 1900s (see Fig 3). This exemption was also widely abused; for example, a 1905 article in a Miami paper [201] reported that one captain “having obtained a permit, captured a manatee…and many are enjoying roast, cutlets, and steaks therefrom,” dryly noting that”science will enjoy the bones later on.” Such abuses eventually provoked backlash; in 1907, the Dade County Commission rejected a permit application because”it was about time to put a stop to the capturing of manatees” [202]. With time, fewer permits were granted, and they often carried stipulations requiring the return to local waters after some defined interval [203]. Later laws, such as a 1939 regulation empowering the Game & Fresh Water Fish Commission to designate and fence manatee breeding grounds, further bolstered protection [204].

Next, the creation of ENP contributed to increases in manatee numbers and the extension of their range to the Gulf Coast, as documented by Moore [23. 24]. Manatees and other rare species were often listed among the rationale for the park’s creation and counted among its major attractions [205, 206]. Conservation officers elsewhere in the state occasionally captured manatees and transported them to ENP for protection [207, 208].

These developments were accompanied by a change in public perceptions of manatees. In the late 1800s and early 1900s, sea cows were often described as “monsters” (e.g., [135]) and frequently misunderstood as “fish” (e.g., [209]) or “amphibians” (e.g., [210]). Over time, their status as herbivorous mammals that nurse and care for their young became more commonly known (e.g., [211]). Manatees became a fixture in popular culture. In the early 1900s, a Miami aquarium advertised captive sea cows among its main attractions, boasting that one was “milked every day at 11 o’clock” [212]. Dimock’s [213] lavishly illustrated account of an effort to capture a manatee for a popular magazine was widely recirculated. Sea cows were ridden in a round-up in Lake Worth [214] and in a water carnival at the Miami Biltmore pool [215]. Protests erupted when manatees were killed, captured, or harassed in St. Augustine in 1920 [216] and in Tampa in 1922 [217]. By midcentury, the hunting of manatees became politically untenable. In 1949, when the Miami Aquarium went in search of a sea cow, the hunt was condemned by the Humane Society and local residents [218], forcing officials to revoke the permits [219]. Similarly, when state conservation agents killed a manatee for a Clearwater aquarium in 1954 [220], a local newspaper declared it a “conservation mockery” [221], citizens flooded newspapers with letters of protest, and the agency head declared they were now “out of the business” of killing manatees [222].

**Fig 5:**
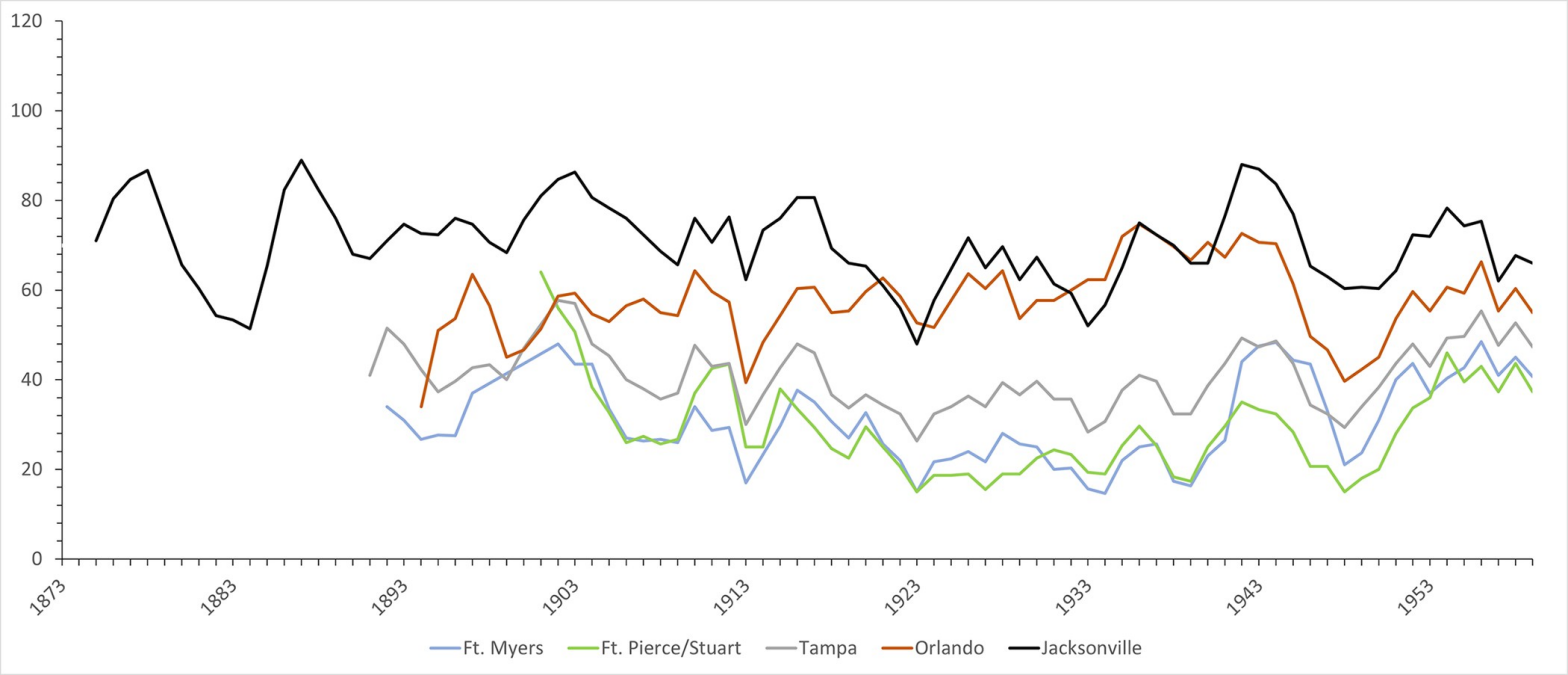


**Fig 6:**
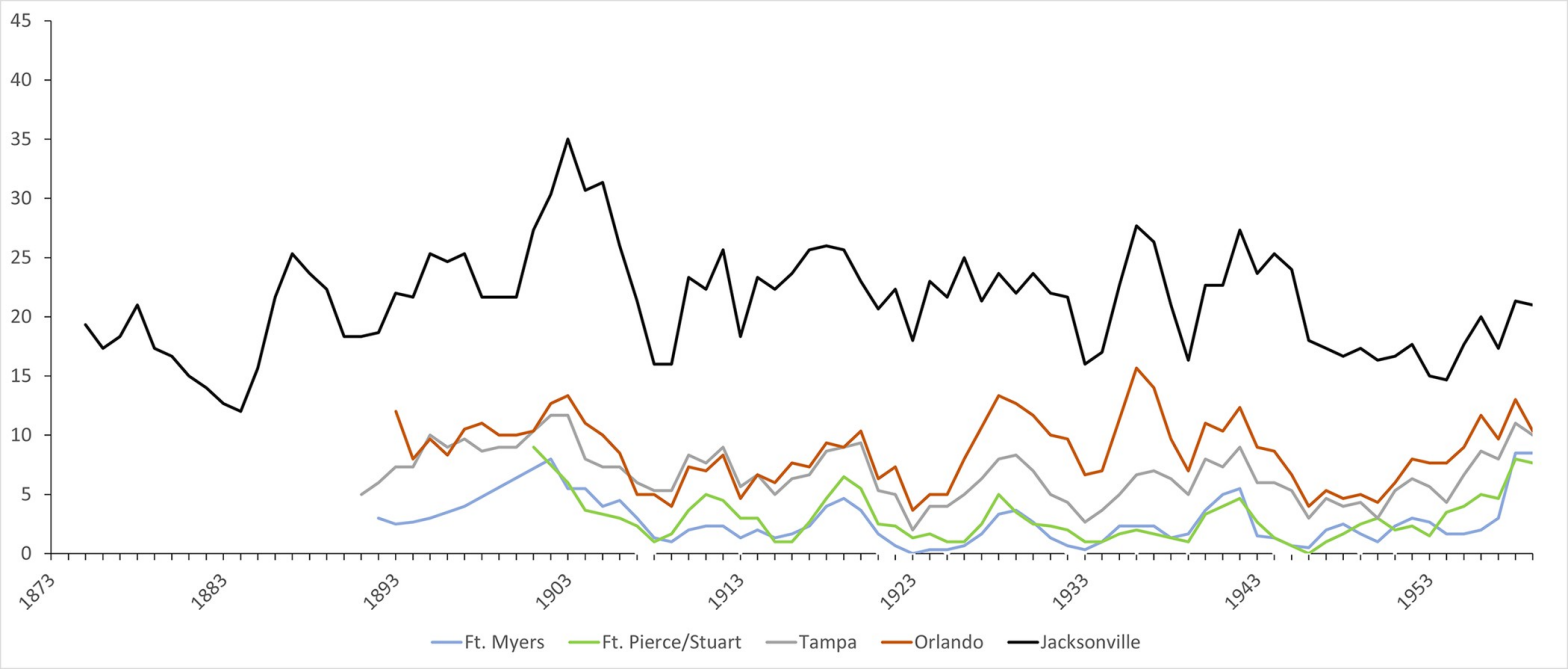


Natural and anthropogenic climate change have also been a critical factor in the increase in manatee numbers and range in the modern era. Historical records from the Florida Keys demonstrate ~0.8°C of ocean warming between the late 1870s and the early 2000s [223], consistent with trends in global temperatures [224]. Analyses of Sr/Ca from corals in the Keys demonstrate that much of this increase in SST was driven by winter warming [225]. Daily temperature records for five Florida cities document a trend toward fewer days per year of potential threat of CSS for manatees from lows or daily averages below ≤9.5°C (Figs 5 and 6). The frequency of days with temperatures cold enough to threaten CSS in manatees appears to have been particularly low between 1904–1909 and 1921–1934, perhaps facilitating range extension northward along the Florida peninsula. However, temperature data also demonstrate the continued threat of CSS for manatees from periodic cold spells. For example, there were more days of potential threat from CSS in 1910 and 1940; cold weather the latter year reportedly resulted in the deaths of several manatees in the Ft. Myers area [226, 227].

**Fig 7:**
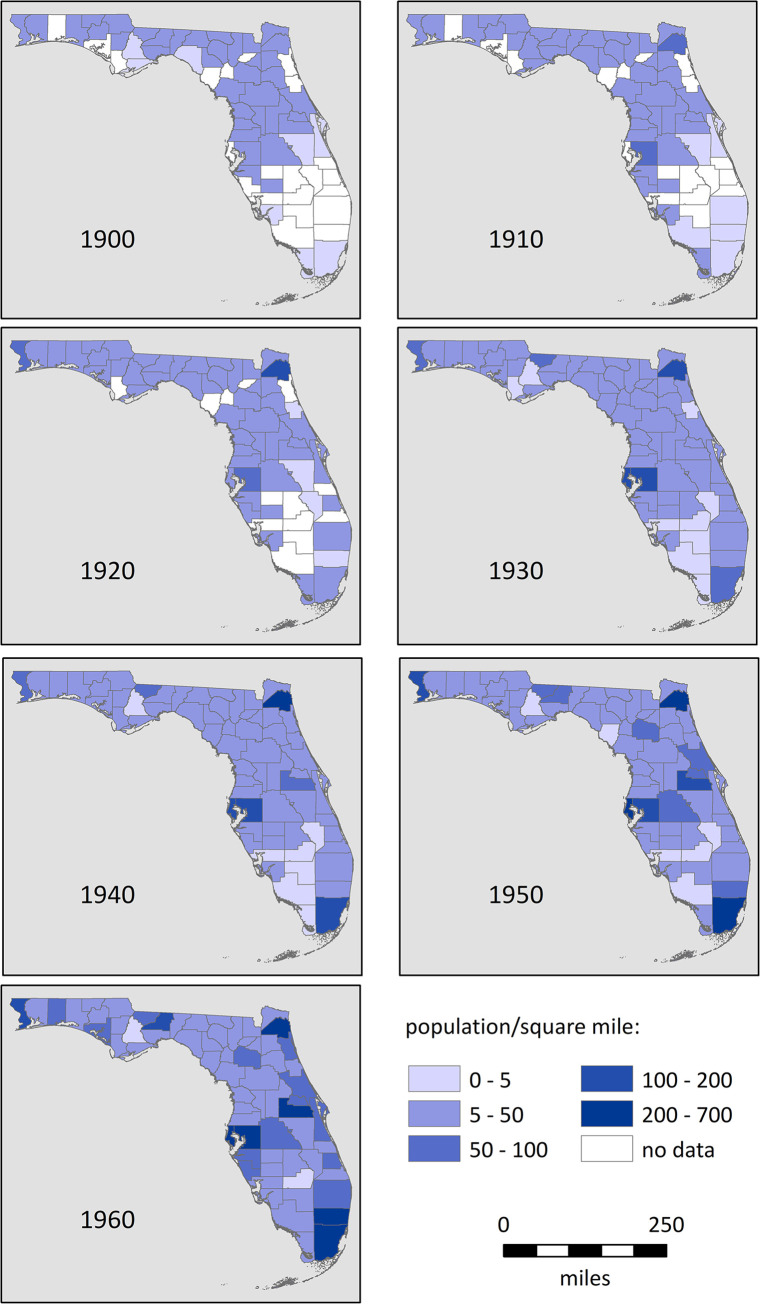


**Fig 8:**
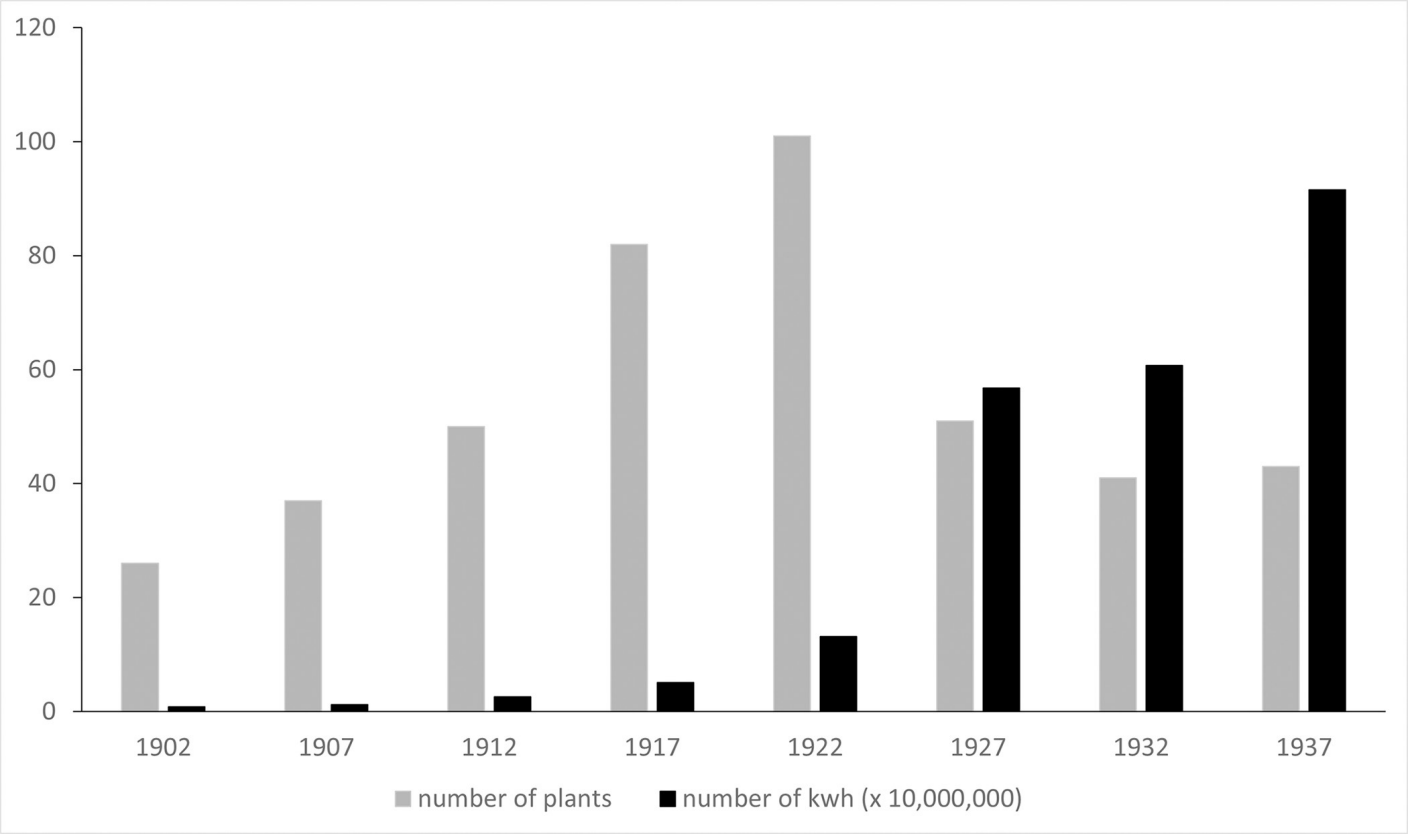


The manatee’s northern expansion was thus reliant on the new forms of warm-water refuges that accompanied modern population growth and development. Indeed, the expansion of human (Fig 7) and manatee populations and ranges in Florida were largely concurrent. In the late 1800s, the human population of southern Florida was low, <5/per sq mi in most counties. By the 1930s, most counties on the Atlantic Coast had grown to >50/sq mi. By the 1950s, most counties on the Gulf Coast also reached this milestone. Many of the reported manatee sightings in the first few decades of the twentieth century were in canals and yacht basins (e.g., [228, 229]. In the 1940s, several articles documented the manatee preference for warm sewage effluent (e.g., [230, 231]. However, power plants soon became the primary form of warm-water refuge. As elsewhere in the U.S. [232], power production in Florida expanded greatly in the first half of the twentieth century (Fig 8). The number of plants increased sharply until 1922; subsequent consolidation resulted in fewer but larger plants with greater productive capacity. The timing of these larger plants is consistent with the manatee’s expansion, first up the Atlantic Coast where plants were constructed in Fort Lauderdale (1926), Palm Beach (1946), and Ft. Pierce (1945), and later up the Gulf Coast to Ft. Myers and St. Petersburg (1958) and Crystal River (1966) [3, 233].

Finally, other anthropogenic changes may have further contributed to the increase in manatee numbers and range over the twentieth century. One major factor may have been the introduction of water hyacinth (*Eichhornia crassipes*) in the late 1800s [234]. The manatee’s preference for this invasive species—sometimes referred to as “manatee manna” (e.g., [235])—has been documented since the 1930s [24, 26, 230, 231, 236].

## Conclusions

Archaeological and historical evidence suggests the Florida manatee has never been as common or ranged as extensively as it does today. Yet even as manatee sightings increased in regularity in the twentieth century, and even as many acknowledged these increases, they were also described as “seldom seen” [237], “becoming scarce” [38]; “almost extinct” [238], “about as extinct as the dodo” [239], or “gone the way of progress” [240], usually under the assumption that this scarcity resulted from recent overhunting. We are not the first to suggest that Florida manatee populations were low before the 1800s (e.g.,[28, 31, 32, 58, 227, 241]), but few studies have considered the extent to which manatees have expanded in number and extent because of human alterations of the landscape, along with natural and anthropogenic climate change, legal protections, and changes in human perceptions.

The fates of humans and manatees in Florida are now thoroughly entangled. In 2021 and 2022, the Florida FWC resorted to feeding lettuce to hungry manatees to compensate for the decline in seagrasses from pollution [242]. The FWC’s latest action plan calls for the retention of industrial sources for warm-water refuge until alternatives can be identified and manatees can be weaned away [5]. Yet many Florida communities are likewise dependent on manatees; around 100,000 people annually visit Crystal River alone to interact with manatees [243] and the economic benefits that sea cows bring to Citrus County (including tourism revenue, jobs, and ecosystem services) are estimated between 8 and 9 million dollars [244].

Kopf and colleagues [21], in their argument for Anthropocene baselines, ask “whether past conditions remain relevant reference points for the restoration and management of contemporary human-altered systems.” The historical ecology of the Florida manatee provides a novel twist on the concept of Anthropocene baselines, however, suggesting that the manatee’s expansion over the last century owes to many of the same factors that now place the species at greater risk. Few would dispute that in this case an Anthropocene baseline is preferable to a historical baseline, if “historical” is conceived as precolonial or pre-modern—and if we are correct in our hypothesis that manatees were exceedingly rare or absent altogether in this interval. There is nothing in the history of manatee expansion across the modern era to argue that their current extent and population levels are necessarily unsustainable, but whether the baseline for manatee management in the future should be their present range and population or those of some earlier point in the Anthropocene is one that may require difficult decisions. Setting appropriate Anthropocene baselines for the Florida manatee may require better understanding of the roles manatees have played, and continue to play, in ecosystem structure and function, to predict the effects that are likely to accompany changes in their numbers and distribution [245]. As one example, we note again that manatees appear to have expanded their range initially through reliance on passive warm-water refuges such as canals and yacht basins. Recent study reinforces the importance of passive thermal basins for manatees in the face of power plant closures [246]. Could existing features of this sort be made more manatee-friendly, or be replicated on a scale sufficient to mitigate the retirement of power plants? As another example, Florida municipalities spend considerable money and effort to eradicate invasive water hyacinth through physical, mechanical, and chemical means, but it may make more sense to manage manatee and hyacinth populations in concert [247–249].

Conservation scientists increasingly recognize that the differential severity of anthropogenic impacts to species abundances and distributions may require that assessments be tailored to the histories of specific subpopulations. Rodrigues and colleagues [19], for example, propose defining different baselines for separate subpopulations of bowhead whales (*B*. *mysticetus*) based on their contrasting histories of exploitation in the modern era. For conservation purposes, the Florida manatee population is currently divided into four broad management units, based on studies of their contemporary distributions, mortality, and threats [250]. It may be advantageous to better incorporate historical data into the definition of these management units, as well as the formulation of assessments for each area.

Wildlife managers face a formidable task in planning future actions to address emerging threats to the Florida manatee, a species that has become among the state’s most famous [138] and most iconic [251] animals. Understanding the manatee’s history is likewise a formidable task, but one that will be important for planning appropriate responses to contemporary challenges. The past remains relevant for “shaping a better Anthropocene” [252] for humans and manatees in Florida.

### Notes

1. This was Robert Osceola, grandson or nephew of the famous warrior [253].
2. Manatee County was given a baby manatee the aquarium had in captivity [254]. Originally referred to as “Stink,” it was re-named “Baby” [255] and later “Snoots” or “Snootie” [256]. It lived in captivity in Bradenton until its accidental death in 2017, at 69 years old [257].
3. Bartram’s [104] mention that the Seminole referred to manatees “by a name which signifies the big beaver” is intriguing, given that beavers were infrequently hunted by Native precolonial peoples of the Southeastern U.S. [258]. However, Bartram also noted that manatees were “counted wholesome and pleasant food” and, as we note, there is evidence of Seminole hunting of manatees in the late nineteenth century. In addition, Indigenous elder Betty Mae Jumber reported that the species is not represented in Seminole legends [259].
4. Zoomorphic forms of ceramic pipes excavated at the Mound City site in Ohio have been identified as manatees [260, 261]. However, the only depicted example [261] has an upright posture and forelimbs with distinct digits, more resembling a monk seal.
5. Alexander Graham Bell also proposed farming manatees to alleviate meat shortages in an article for a scientific journal [262] that was widely redistributed in the popular press.

## Supporting information

S1 Dataset Systematic and opportunistic archaeological and archival samples. (XLSX)
